# COVID-19 and the teacher's voice: self-perception and contributions of speech therapy to voice and communication during the pandemic

**DOI:** 10.6061/clinics/2021/e2641

**Published:** 2021-03-19

**Authors:** Katia Nemr, Marcia Simões-Zenari, Vanessa Cássia de Almeida, Glauciene Amaral Martins, Isabele Tiemi Saito

**Affiliations:** Departamento de Fisioterapia, Fonoaudiologia e Terapia Ocupacional, Faculdade de Medicina FMUSP, Universidade de Sao Paulo, Sao Paulo, SP, BR.

**Keywords:** Voice, Voice Disorders, Voice Quality, Voice Training, School Teachers, Occupational Health

## Abstract

**OBJECTIVES:**

We aimed to analyze the vocal self-perception of Brazilian teachers and their communication needs, vocal signs and symptoms, and voice-related lifestyles during the coronavirus disease (COVID-19) pandemic and, based on this information, to develop guidance materials intended for dissemination to these teachers and the general community.

**METHODS:**

An online questionnaire designed for this survey was distributed *via* the researchers' networks and was available for completion by any teacher, except those who were not working at the time. There were 1,253 teachers from all over Brazil, of both sexes, covering a wide age range, working at different levels of education, and most with more than ten years of experience. Descriptive and inferential analyses of the data were performed.

**RESULTS:**

On comparing the prepandemic period with the current one, participants indicated voice improvements. In contrast, they presented symptoms such as dry throat, effort in addressing remote classes, hoarseness after classes, and difficulties with the use of headphones, among others. They further indicated stress, general fatigue, impact of the pandemic on mental health, and the overlapping of many home tasks with professional tasks. Some smoked, and others hydrated insufficiently.

**CONCLUSION:**

Although teachers generally noticed voice improvements during the pandemic, a proportion of them perceived worsening of voices. Many indicated several factors in which speech-language pathologists could guide them with the aim of improving performance and comfort during remote and hybrid classes, an initiative that will positively impact not only their voice and communication but also their quality of life.

## INTRODUCTION

In December 2019, the world was alarmed by the transmission of the novel severe acute respiratory syndrome coronavirus 2 that was identified in Wuhan, China and caused coronavirus disease (COVID-19), which spread through transmission from person to person. It presents a clinical spectrum ranging from asymptomatic to severe infection (1).

The biopsychosocial effects of this pandemic and its consequent impact on the general health of the population have motivated researchers to pursue knowledge regarding the different scenarios in recent months.

New research has emerged daily in several areas, such as health, economy, and education, among others. In terms of health, studies initiated in hospitals have investigated the complications and therapeutic results of infected patients, and currently, literature reviews and/or meta-analyses have started to emerge from the published data (2,3). The physical, mental, and emotional impacts on health professionals who work with these patients have also been investigated (4,5).

Experts from all continents have dedicated themselves to understanding the consequences of the new scenarios imposed by social isolation or distancing. In this new reality, hybrid contexts, in which some activities can be implemented in person and others at a distance, have become common.

This context has not been different in the area of speech therapy, and recent research on voice has been dedicated to understanding the following: professional vocal use during this pandemic period, especially in singers (6); the impact of wearing facemasks (7); the risks of vocal changes in different professionals who are working remotely (8,9); and the challenge of virtual voice therapy (10,11).

The teacher's voice has been one of the themes most researched by specialists for decades, especially regarding symptoms and risk factors. However, there is much to understand regarding the dynamics of this worker. Owing to the pandemic, there has been a drastic change in their work activities and communication.

The impact of this new professional reality on the voices of teachers at different levels of education remains unclear. A study involving 313 university professors showed that the psychological stress surrounding the transition to online, synchronous teaching has been associated with high levels of vocal symptoms (12).

Before the pandemic, specialists made an effort to understand teachers' communicative demands and factors related to the presence of dysphonia as well as to develop interventions that would help them with the use of their voices. In this peculiar period the world is going through, there is a need for even greater effort.

The objective of this study was to analyze the vocal self-perception of Brazilian teachers from varying levels of education and different regions of the country as well as their communication needs, vocal signs and symptoms, and voice-related lifestyles during the pandemic. Based on this preliminary information, we propose emergency support material intended for these teachers and the community in general.

## METHOD

This research aimed to present preliminary data from stage 1 of this transversal, prospective, observational research involving teachers from both public and private educational institutions as well as autonomous teachers. The research was approved by the Research Ethics Committee of USP Hospital das Clínicas da Faculdade de Medicina da Universidade de São Paulo (HCFMUSP; CAAE 32876820.8.0000.0068; study protocol number 4.127.673). The participants completed a free and informed consent form (TCLE).

Participating teachers were of any age and sex, and they were working in different levels of education (early childhood education, elementary school I and II, high school, and university), teaching different subjects (generalist or specialist), involved in remote activities at the time of data collection, and/or engaged in private classes.

The exclusion criteria were as follows: retired teachers or those not teaching during the period of social distancing caused by the COVID-19 pandemic.

The questionnaire (Google Forms) was sent to the authors' contact networks by e-mail and mobile phone; posted in social networks; distributed through various media, such as newspapers, radio, and websites; and shared with institutions that engage in teacher training. In this questionnaire, the teacher could choose whether or not to receive informative material regarding the use of his/her voice at work.

Teachers who received the questionnaire could freely pass it on to other professional colleagues.

The collection period was between July and October 2020.

In the first stage of the study, 1,493 teachers completed the questionnaire, and 240 were excluded for incomplete or incorrect data, duplicate data, or non-consent indicated in the TCLE.

The final sample for this analysis comprised 1,253 teachers, including 1,025 women and 228 men, with ages ranging from 18 to 78 years (mean, 43.14 years; standard deviation, 10.76 years).

The performance time varied from 1 to 52 years (mean, 16.15 years; standard deviation, 10.20 years). Of the workplaces involved, 679 (54.2%) were in public education institutions, 411 (32.8%) in private institutions, 94 (7.5%) self-employed, and 69 (5.5%) in public and private schools.

The participants were from 21 Brazilian states, distributed throughout the five regions of Brazil, and many were in more than one level of education: 695 (33.0%) in elementary school I, 411 (19.5%) in elementary school II, 345 (16.4%) in early childhood education, 345 (16.4%) in high school, 212 (10.0%) in university, and 98 (4.7%) in other types of education (private classes, academies, technical education, and postgraduate). Among the participants, 1,081 (86%) opted to receive informative material on voice and communication.

For this preliminary analysis, the following items were considered: self-perception in relation to their voice before and during the current pandemic as well as other variables; vocal signs and symptoms, such as hoarseness, dry throat, sore throat, air in the voice, tiredness in the voice, and voice failures; hydration; coffee drinking; smoking; hours of sleep; whether it was necessary to make efforts to communicate in virtual classes and meetings; if there was difficulty conducting remote classes; if changes had occurred in terms of voice changes or changes in vocal habits; use of headphones; if there was something that made the voice worse or better; tiredness, fatigue, or exhaustion; alcohol consumption and use of cigarettes and/or other drugs; health issues; diagnosis of COVID-19; nonprofessional activities during the pandemic and their consequences on the body; and mental and emotional self-perception.

Vocal self-perception during both periods was based on the following classifications: lousy, bad, reasonable, good, or great.

The comparison between responses regarding the prepandemic period and those regarding the current pandemic period was performed using the Wilcoxon signaled posts test. Other variables were descriptively analyzed.

While the data collection at this stage of the research took place, the first video was produced and sent to the participants who opted to receive informative material as soon as the completion of the questionnaires was accomplished. In this video, general aspects of teacher communication during face-to-face classes were deliberated (13,14), with emphasis on voice and expressiveness, environmental factors, how the voice is produced, how communication occurs during remote classes, and factors that interfere negatively and positively with communication. Literature on the subject and the authors' experience were considered a basis (first video available: https://youtu.be/kZQc7GbJ1X4).

The development of the second video will be guided by a descriptive analysis of the questionnaire responses and comparison with material covered in the first video. The intention is to advance and to deepen their self-perceptions. These data will be presented as a script for the second video to optimize the use of voice and communication in this period.

## RESULTS

### Self-assessment of voice quality and self-perception of factors that positively and negatively impact voice and communication

Vocal self-perception improved during the current pandemic compared with that during the prepandemic period (Table 1).

Approximately 30% of the participants noticed an improvement in voice, while 150 (12%) indicated a worsening, and the remaining 734 (58%) did not experience any change.

**Figure 1:**
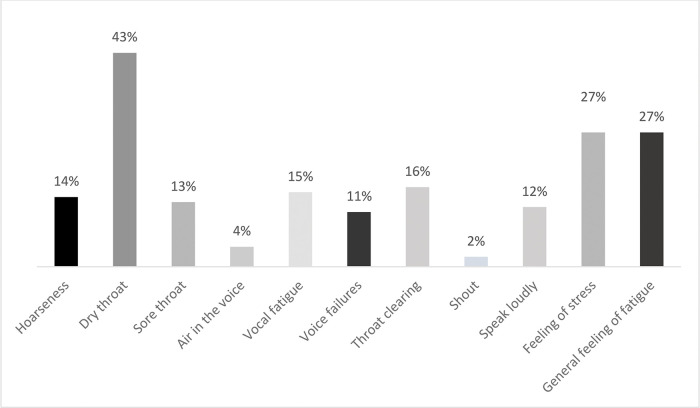
Distribution of vocal and nonvocal signs and symptoms and voice usage during the current COVID-19 pandemic as reported by participants.

The most commonly cited symptoms and vocal habits during the current pandemic were dry throat (N=539, 43%), stress (N=338, 27%), and general feeling of tiredness (N=338, 27%) (Figure 1).

**Figure 2:**
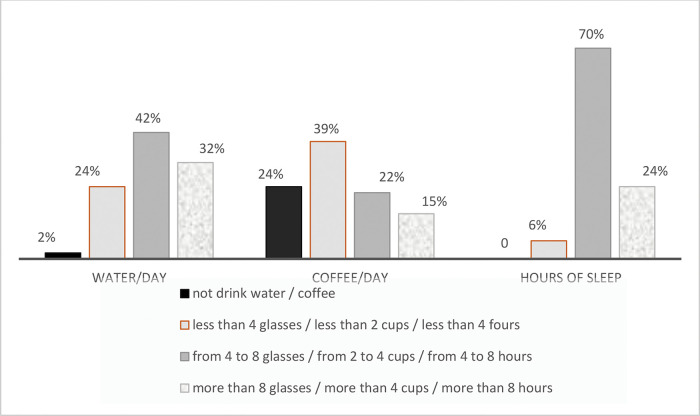
Distribution of data regarding water and coffee consumption and sleep duration (hours) during the current COVID-19 pandemic.

Participants indicated more frequent drinking of four to eight glasses of water per day (N=526, 42%), up to two cups of coffee per day (N=488, 39%), and sleep durations of between 4 and 8h per day (N=877, 70%) (Figure 2). Approximately 75 (6%) mentioned tobacco smoking, in a range of 1 to 40 cigarettes per day.

Half of the teachers reported making more vocal effort during remote classes/meetings; of these, 825 (65.8%) believed that this greater effort negatively impacted their professional performance.

Difficulties caused by remote classes were indicated by 203 (17.6%) participants, with the following being the most cited: hoarseness, vocal fatigue, non-satisfaction with vocal quality, voice failures, sore throat, dry throat, throat clearing, and difficulties with technology.

Changes in voice during the pandemic were reported by 307 participants (24.5%); of these, 845 (67.4%) indicated that their voices changed for the better and 408 (32.6%) for the worse.

Factors indicated by 407 participants (32.5%) to have improved their voices during the pandemic were mainly resting/talking less/being silent and drinking water. Factors that made the voice worse were indicated by 286 participants (22.8%) and mainly included talking a lot, stress/nervousness/tension/anxiety, and the use of headphones.

New habits (positive or negative) were cited by 249 participants (19.9%), and these included controlling speed/vocal intensity, drinking more water, modifying the diet, vocal exercises, self-medication, or home remedies for the voice (ginger, salt, and vinegar, among others), vocal therapy, singing classes, meditation/physical exercises, reducing stress/anxiety, interrupting vocal exercises, reducing the ingestion of water, increasing vocal use, and using headphones and microphones, among others.

A total of 801 teachers (63.9%) indicated the use of headphones for remote communication.

Approximately 25% of the participants cited conditions that worsened their voices, and these included prolonged voice use, vocal effort, climate variation or low air humidity, anxiety/stress/irritating/fear, online classes/meetings and use of technology, breathing issues, lack of auditory and visual feedback or student interaction, dust, use of the mask, low water consumption, inadequate posture and tension, tiredness, noise, smoking, wearing headphones, talking while sitting, staying at home, mold/humidity, sore throat, no vocal exercises, inadequate diet, coffee, and cleaning products, among others.

In contrast, 446 (35.6%) teachers indicated that the following factors improved their voices: reducing vocal use, drinking water, avoiding vocal effort, voice rest, adequate diet, home remedies for voice symptoms (ginger, salt, and vinegar, among others), vocal exercises, moments of rest/leisure, noise control, working in an adequate environment, good quality sleep, reducing stress, voice therapy, using a headset/microphone, having fewer students, and drinking hot coffee, among others.

A total of 254 participants (20.3%) indicated feelings of vocal fatigue, tiredness, and exhaustion at the end of a day full of classes and meetings, 142 (11.3%) at the end of a class or virtual meeting, and 139 (11.1%) at the end of a week with many classes and meetings.

Most participants (44.3%) did not consume alcoholic drinks, cigarettes, and/or other drugs; on the other hand, 204 (16.3%) indicated that this consumption increased during the pandemic.

**Figure 3:**
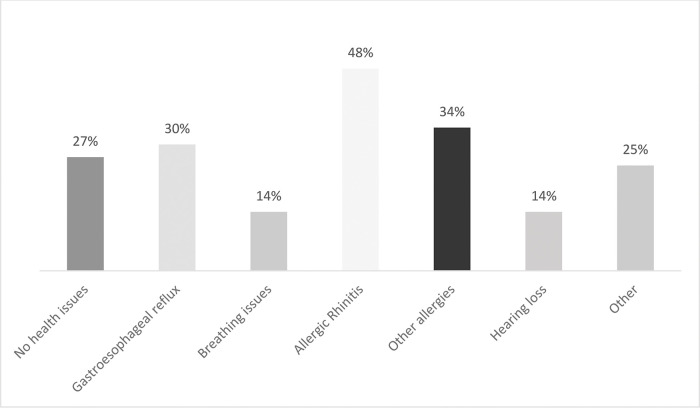
Distribution of data on health issues during the current COVID-19 pandemic.

When asked about health issues during the pandemic, allergic rhinitis, other allergies, gastroesophageal reflux, and respiratory issues were the most cited (Figure 3).

Thirty-one (2.5%) teachers were diagnosed with COVID-19. Of these, 12 (38.7%) reported that their voices were altered because of the disease, 7 (22.6%) still had respiratory issues after the disease, 2 (6.5%) were hospitalized, and none required intubation.

**Figure 4:**
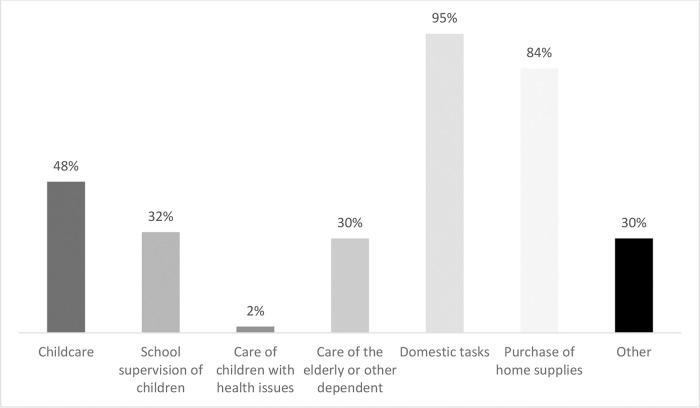
Distribution of data on household activities performed by participants during the COVID-19 pandemic.

In relation to nonprofessional activities during the pandemic, responsibility for the care of the home/domestic chores, purchases of home supplies, and caring for children were highlighted (Figure 4).

According to the teachers, these activities contributed to an increase in stress (N=556, 44.4%), body/muscular fatigue (N=538, 42.9), mental fatigue (N=525, 41.9%), and vocal fatigue (N=117, 9.3%).

Among the teachers, 954 (76.1%) believed that the pandemic was affecting their mental health.

### Aspects of voice and communication to be addressed in the second video

Based on voice and communication data submitted by the teachers in relation to the current pandemic period, it was possible to define the priority items to be considered in the second video as they were not addressed in the first video or because of the need for detailing:

1. Self-perception: what would be a “good voice” for a teacher;
2. Symptoms: dry throat as a highly occurring symptom and its relationship with low hydration, presence of dust, self-medication, allergy, cleaning products, smoking, and the way forward;
3. Vocal effort during vocal symptoms after online classes/meetings: reinforcement regarding attention to negative habits such as throat clearing, very strong speech, inadequate posture, and importance of voice rest;
4. General stress and tiredness and its relationship with professional vocal demand, vocal demand outside of work, and other activities at home;
5. Factors worsening the voice: lack of scientific evidence supporting the use of home remedies for the voice as well as voice and general health risks due to increases in alcohol/drug consumption and smoking;
6. Factors improving the voice: maintenance of voice therapy for those under treatment, importance of vocal exercises as well as vocal warm-up and cool-down for those having this orientation, vocal rest that can be associated with physical or leisure activities, guidance on the proper use of headphones and facial masks during vocal activities, and taking care of health issues and the sequelae of COVID-19.

## DISCUSSION

This study sought to investigate the impact on voice and communication in Brazilian teachers made by the necessary changes in teaching because of imposed social isolation intended on containing the COVID-19 pandemic.

It is believed that the research had expressive repercussions, with a scope that allowed the representativeness of all regions of the country, considering the participation of teachers from 21 Brazilian states. Another interesting point was the total number of men participating. Although studies with professors have had a greater participation of women, with the average percentage of participating men corroborating that in the present study (*i.e.*, approximately 20%) (15,16), the absolute number exceeds that in most other studies in this area. A possible justification for this high number of male participants would be the application of broad inclusion criteria, in which all levels of education and independent teachers were considered.

This enabled better analysis in relation to sex, in addition to knowledge regarding the specific demands of male teachers and continuity of data analysis. Notwithstanding, because of the greater representation of women in this research, as observed in most studies on the teacher's voice, the data is comparable to that of other studies on this subject.

Likewise, the age range studied was extensive and potentially allows cuttings with specific analyses regarding elderly teachers.

In addition, public and private schools, independent professionals, and different levels of education were represented in this study. Most participants had more than ten years of teaching experience.

It was observed that social distancing and work changes during the pandemic had an impact on voice improvement according to teachers' self-perceptions. These data can possibly be explained by teachers’ distance from the physical work environment and reduced contact with the different risk factors inherent to the profession, such as dust, noise, and exposure to violent situations, among others (17). Additionally, these data could also be attributed to lack of exposure to other aspects that can impact their general health, such as daily displacements, which often expose people to hours of heavy traffic in large cities. Thus, whereas the COVID-19 pandemic has ushered in adversities and changes, the school environment on the other hand, that is, during face-to-face classes potentially impacts teachers' voices more negatively according to their own perception.

However, it is important to highlight the significant number of teachers who perceived a worsening voice during the pandemic. It is necessary to analyze whether this group includes those who were diagnosed with COVID-19 or if it was more difficult for them to adapt to distance learning and to establish the factors that contributed to this worsening of the voice.

Although most teachers perceived voice improvements, the dry throat symptom was a common occurrence during the pandemic period and could have been related to hydration, allergies, inadequate feeding, self-medication, and exposure to dust and cleaning products. Dry throat is one of the most common symptoms among teachers (16,18,19). In this study, it was mentioned that even when the voice was considered to have improved, other aspects led teachers to consider this improvement.

Data from other studies have implicated psychosocial aspects. A recent study analyzed the responses to an online questionnaire completed by 313 university professors in Israel at the end of the first week of online synchronous teaching during the COVID-19 pandemic. As per their observations, the authors considered it to be the most overloaded and stressful week of the transition. This psychological stress was associated with high levels of vocal symptoms, especially in those who reported high levels of psychological stress in the periods before the pandemic (12). The notion that psychological stress can have a negative impact on the voice is a relevant theme and has been described in the literature (13,16). The emotional impact must be carefully analyzed, especially in this exceptional era that the world is experiencing, to understand if there was a period during these months when it was more prominent according to the teachers' perceptions. Overload because of the accumulation and overlapping of professional and home-based tasks may contribute to the stress and general tiredness mentioned by the participants. In addition, more than 16% reported an increase in the consumption of alcoholic beverages, smoking, and/or use of other drugs, which should be considered not only as risk factors for voice worsening but also for the emergence of other diseases, such as gastroesophageal reflux (20) and head and neck cancer (21).

Several aspects related to voice and communication were mentioned, such as vocal effort during online classes, hoarseness and vocal fatigue after vocal demands, difficulties with wearing headphones, and speaking wearing a mask, among others, all of which are included in the script of the second video that will be produced, distributed to participants, and availed to the community. It is important to highlight that teachers often mention behaviors that are not supported by scientific evidence or that may even worsen the voice, such as home remedies for the voice using ginger, salt, vinegar, and hot coffee. A discussion on aspects supported by scientific evidence of improvement and those that potentially harm the voice is also being considered in the video script. Additionally, the important aspect of communication in the hybrid format of classes, that is, the often simultaneous face-to-face and online instruction, will be addressed, as it is a model that has begun to be adopted as institutions return to face-to-face classes and should be a common scenario in the following months in Brazil and other countries.

It is important to consider that a recent meta-analysis showed that sex, upper airway issues, caffeine consumption, speaking loudly, number of classes per week, and the experience of dismissal because of voice issues were the main risk factors for teachers' voice disorders. In contrast, age, number of students in class, consumption of alcoholic beverages, physical activity, smoking, water consumption, singing habits, duration of teaching, perception of noise inside the school, number of classes per day, evaluation of noise inside the classroom, and perception of technology and instruments inside the workplace were not related to the presence of voice disorders. However, the author encourages further research to estimate the risk factors for voice disorders in teachers based on the results yielded (22).

Among the teachers who had COVID-19, many mentioned that their voices were altered after the disease. It would be important to follow them up in relation to respiratory sequelae and other yet unknown eventualities, including possible repercussions on the voice.

Based on the data analyzed, it was possible to prepare the script for the second informational video on voice and communication in education. It was realized that it would be possible to deepen the content of various aspects covered in the first video. In addition, guidelines will be introduced that involve the wearing of masks during classes as a result of hybrid teaching (*i.e.*, concurrent face-to-face and online instruction), which is a reality for many teachers at present. Furthermore, questions related to the possible negative impact of COVID-19 on vocal production are yet to be addressed.

The continuity of this research foresees the remote recording of the voices of interested teachers, with appropriate technological support. Thus, it will be possible to analyze vocal characteristics (23) that will allow new orientations for these teachers and afford a better understanding of the relationship between the voice and several variables of interest.

The data also reinforce the need for multivariate analysis mainly because dysphonia is a multifactorial disease. The possible effects of videos with voice and communication information can also be studied in the future.

Previous vocal training, orientation, and/or treatment were not investigated in the present study, and these aspects may influence the use of teachers' voices and communication in online classes. These factors can be considered in other studies.

Furthermore, a more detailed future analysis should consider the date on which the participant filled out the questionnaire, as our collection period was between July and October 2020, subjecting the results to different phases in the advancement of the pandemic. Therefore, experience with remote work varied among teachers, which may have interfered with the findings and needs to be considered, since the impact of the pandemic may have been greater during its initial stages.

## CONCLUSION

Although teachers generally perceived improvements in their voices during the pandemic because of being distanced from several factors that could contribute to the occurrence of dysphonia, a proportion of them perceived worsening voices. Many indicated several factors in which speech-language pathologists can contribute to guiding them, with the aim of improving performance and comfort during distance and hybrid classes, which will impact not only their voice and communication but also their quality of life.

## AUTHOR CONTRIBUTIONS

Nemr K, Simões-Zenari M were responsible for the study conception and design, data acquisition, analysis and interpretation, manuscript drafting, revision for key intellectual content, and approval of the manuscript final version for submission. Almeida VC, Martins GA and Saito IT were responsible for the data organization, support in the data analysis, and approval of the manuscript final version for submission.

## Figures and Tables

**Table 1:** Comparison of periods before and during the pandemic in relation to vocal self-perception.

			During												
			Lousy		Bad		Reasonable		Good		Great		Total		
Variable		Classification	n	%	N	%	n	%	n	%	n	%	n	%	*p*
Vocal self-perception	Before	Lousy	3	0.24	7	0.56	7	0.56	7	0.56	1	0.08	25	2.00	<0.001*
		Bad	**4**	**0.32**	18	1.44	92	7.34	36	2.87	11	0.88	161	12.85	
		Reasonable	**5**	**0.30**	**38**	**3.03**	321	25.62	147	11.73	29	2.31	540	43.10	
		Good	**1**	**0.08**	**6**	**0.48**	**63**	**5.03**	339	27.06	32	2.55	441	35.20	
		Great	**0**	**0.00**	**0**	**0.00**	**8**	**0.64**	**25**	**2.00**	53	4.23	86	6.86	
	Total		13	1.04	69	5.51	491	39.19	554	44.21	126	10.0	134	100	

Wilcoxon signaled posts test.

Legend: *: statistically significant value=5% (*p*≤0.05).

Note: Medium-gray shaded cells indicate individuals who did not migrate from their prepandemic category during the pandemic; the light-gray shaded cells indicate individuals who migrated to better vocal quality categories during the pandemic; and dark-gray shaded cells indicate individuals who migrated to worse vocal quality categories during the pandemic.
